# Measuring empathy in OSCEs: A comparison of student, standardized patient, and observer rating tools

**DOI:** 10.1016/j.pecinn.2025.100452

**Published:** 2025-12-29

**Authors:** A. Hepner, B. Cerutti, R. Lüchinger, N. Junod Perron

**Affiliations:** a Geneva Faculty of Medicine, Geneva, Switzerland; b Unit of Development and Research in Medical Education, Geneva Faculty of Medicine, Geneva, Switzerland; c Medical Directorate, Geneva University Hospitals, Geneva, Switzerland

**Keywords:** Behavioral empathy, Correlation, Perceptions, Student, Simulated patient, Observer, OSCE

## Abstract

**Aim:**

During objective structured clinical examinations (OSCEs), perceived empathy can be rated by medical students, simulated patients (SPs) or external observers. This study aimed to examine how different ratings of medical students' perceived empathy correlate with each other, as well as with measures of behavioral empathy using a standardized coding system.

**Methods:**

Voluntary medical students who participated to a formative OSCE self-rated their empathy using a global empathy score. SPs and an external observer rated students' perceived empathy using the Jefferson Scale of Patient's Perceptions of Physician Empathy (JSPPPE-Patient; JSPPE-Observer) and global scores. The Verona Coding System (VR-CoDES) captured patients' emotions and students' responses (verbal empathy). Non-verbal behavior and five sub-behaviors were assessed via an ordinal scale (1–5). Associations were calculated using correlation analyses.

**Results:**

56 medical students participated. There was no significant association between students' self-perceived empathy and other measures of perceived empathy. Global SPs' and observer's measures of perceived empathy as well as JSPPPE scales correlated with verbal empathy (VR-CoDES) (global ρ =0.27 *p* < .05; respectively 0.56, *p* < .01), JSPPPE-P (ρ = 0.34, p < .05), and JSPPPE-O (ρ = 0.41, *p* < .01). Non-verbal behavior correlated with global scores (SP ρ = 0.39 *p* < .01; observer ρ = 0.66, p < .01) and verbal empathy (VR-CoDES) (ρ = 0.38, p < .01).

**Conclusion:**

SPs and observers' ratings of perceived empathy using global scores as well as measurement of non-verbal behavior are valid ways to assess medical students' behavioral empathy.

**Innovation:**

Standardized coding systems are useful to assess which measures of medical students' perceived empathy used in OSCEs best correlate with behavioral empathy.

## Highlights

• Students’ self-rated empathy does not align with perceived empathy ratings from simulated patients or external observers.
• Simulated patient and external observer assessments of empathy are correlated.
• Student non verbal behavior ratings correlate with verbal empathy and perceived empathy from simulated patients and observers.

## 1 Introduction

Empathy is considered to be crucial for the delivery of high-quality care since it plays an important role in establishing trust in patient-physician encounters. It helps to build trust and reduces patients' anxiety and feelings of distress [1]. Ultimately, empathetic communication improves patient outcomes, health, and compliance [2,3]. Although there is no clear definition of empathy [4], it is commonly described as a multidimensional construct including cognitive, affective, and behavioral dimensions. Cognitive empathy consists of recognizing and understanding a person's perspectives and feelings [5]. Affective empathy is defined as being emotionally sensitive and concerned for another person [6]. Behavioral empathy includes verbal and non-verbal expressions of understanding, respect, and support for another person [7]. Some authors add a fourth dimension, moral empathy, a concept close to compassion, which is an internal motivation of concern for other, and a desire to relieve their suffering by caring and driving acts of altruism.

Measuring and assessing empathy can be challenging because of its complexity and its multidimensional construct. Cognitive and affective empathy is essentially measured through self-questionnaires such as the Jefferson Scale of Physician Empathy [8] or the Interpersonal Reactivity Index (IRI) [6]. Behavioral empathy which refers to observable actions of empathy in practice can be measured through different perspectives: self-evaluation by students themselves, by patients (simulated or real), or external observers [[9], [10], [11]]. Behavioral empathy can be measured via structured observation using either global rating tools or scales, checklists that pay attention to both verbal empathy and non-verbal behavior. Non-verbal behavior can be further split into five sub-dimensions: eye contact, tone of voice, head nods, gesture, and the postural position, which can be measured separately [12]. Finally, dyadic coding systems, that break down conversations into coded units, can be used to assess empathy by analyzing how emotional communication (and response to it) unfolds between patients and students [13]. Unlike global ratings or checklists, dyadic coding analyzes what emotions patients express and whether and how students respond to them and uses standardized definitions.

Few studies have examined the association between students', patients', and observers' respective ratings regarding empathy during OSCEs and the results vary [4]. Even less studies have used verbal measures of behavioral empathy using dyadic standardized coding systems and explored how these measures that rely on clear definitions of emotions and responses to them relate to global perceptions of empathy [14]. Though, this is of importance since empathy and more generally communication is often measured using self-perceptions or examiners' ratings. Furthermore, the association between patient-rated and external observers' behavioral empathy is not clear [15]. External observers are not part of the encounter and what they rate is more the conveyance of empathy than the experience of empathy. The aim of this study was to explore which measures of medical students' perceived empathy correlated with each other. We were especially interested in analyzing the association between global ratings of perceived empathy by medical students, SPs and an external observer, and measures of behavioral empathy (verbal empathy and non-verbal behavior) using a standardized coding system.

## 2 Material and method

### 2.1 Design and setting

This study took place at the Faculty of Medicine of the University of Geneva, Switzerland in 2022. This Faculty offers a six-year curriculum (three pre-clinical years and three clinical years) to ca 160 medical students per year. Communication skills training (ten two-hour seminars) on basic skills, including empathy, takes place during the second and third pre-clinical years. During clinical years, students rotate through various clinical clerkships of one or two months in the different fields of medicine and follow seven two-hour seminars regarding more complex communication issues. The undergraduate medical training concludes with a national licensing exam, which consists of two sessions of MCQ written exam and twelve OSCE stations in which communication is assessed.

### 2.2 Participants

Fourth year medical students (1st year of clinical clerkship) were invited by email to participate to a formative OSCE at the beginning of a two-month clerkship in primary care. Sixth year students (3rd year of clinical clerkship) were invited by email to take part into a formative OSCE at the end of their training, one month before the licensing exam (August 2022). All participating students received information and gave a written consent to the study. The study project was approved by the University of Geneva's Ethics Committee (CUREG-MM-2022-01-07).

### 2.3 Development of formative OSCE

NJP created four clinical cases: two cases were designed for fourth year students' level (subacute abdominal pain and thoracic pain), and two other cases for sixth year students (subacute dizziness and headache). All four cases shared similar patient's socio-professional characteristics, levels of worries, and sources of stress (Appendix A).

Five female simulated patients (SPs) attended a two-hour training before every formative OSCE, to simulate a 28 to 35 years old patient (a single scenario per patient) and responded in a standardized way to students' queries. A special emphasis was put on the number of cues and concerns they were expected to mention, and the way they expressed them while describing their symptoms.

### 2.4 Procedure

The students attended the 18-min formative station. The student-SP interaction was filmed. Two investigators (AH and NJP) also observed the OSCE through the tinted glass to check the SP's performance and give oral feedback, if desired, to the participants for learning purposes. Students and SPs completed an on-line self-administered questionnaire with a tablet (Qualtrics) after the formative OSCE [16].

### 2.5 Outcome measures

#### 2.5.1 Global empathy

*Students*' self-perceived global empathy score was measured using a 1–10 ordinal scale (“How would you rate the empathy (verbal and non-verbal) you expressed toward the patient? (circle the corresponding number)”). *SP*s' perceptions of students' overall empathy were measured using a global empathy score (1–10 ordinal scale), and the Jefferson Scale of Patients' Perceptions of Physician Empathy (JSPPPE-P) (5 items- 1-7 Likert scale) using a French valid version in real time [5]. The perceptions of an e*xternal observer* regarding students' overall empathy were measured in the same way as for *SPs*, using a global empathy score (1–10 ordinal scale), and the Jefferson Scale of Patients' Perceptions of Physician Empathy (JSPPPE-O) (5 items- 1-7 ordinal scale) at distance from the OSCE and based on recordings. The external observer (NJP) was an experienced communication skills teacher of the Faculty of Medicine of Geneva.

#### 2.5.2 Behavioral empathy

Behavioral empathy included both verbal empathy and non-verbal behavior.

- Verbal empathy

Verbal empathy was assessed using the Verona Coding Definitions of Emotional Sequences, a dyadic coding system developed by the Verona Network on Sequence Analysis [17,18]. This system is used to single out concerns (defined as: “A clear and unambiguous expression of an unpleasant current or recent emotion where the emotion is explicitly verbalized”) and emotional cues (defined as: “A verbal or non-verbal hint which suggests an underlying unpleasant emotion and would need a clarification from the health provider”). The response to these concerns and cues by the students/physicians is coded (Appendix B). Students' verbal empathy can be specifically considered when the student explicitly acknowledges the affect related to SPs' cues and concerns, explicitly expresses empathy, or acknowledges and legitimate SPs' cues and concerns or emotions in an implicit way [19]. It included the following categories of the Verona coding: non-explicit acknowledgement, non-explicit implicit empathy, explicit affect acknowledgement, and explicit affect empathy.

- Non-verbal behavior

Non-verbal behavior was also measured using the Verona coding manual: it considers five non-verbal sub-behaviors that we assessed using a rating scale (Likert scale 1–5) [20]: eye contact, facial expression, head movement, bodily posture, and tone of voice.

The filmed student-SP interactions were coded using the “The Observer” software [21].

### 2.6 Analysis

#### 2.6.1 Coding

Verona coding: NJP and AH first coded two videos together, then eight videos separately, followed by a comparison of their respective coding to calibrate their coding. Cohen's Kappa coefficients, used to check inter-coder reliability on a subsequent sample of five videos, were excellent: 0.87 for cues/concerns and 0.80 for the students' responses. Non-verbal behavior: after a calibration process, the estimated intercoder reliability, based on ten videos, was also good (Cohen's Kappa = 0.87).

#### 2.6.2 Statistical analysis

We analyzed the frequencies of the emotional cues and emotions expressed by the SPs and the students' responses coded on “The Observer”. Correlations between the global empathy scores (self-evaluation, by the SPs and the observer), the verbal empathy scores obtained with Verona coding manual, and the non-verbal behavior scores, were estimated using Spearman's rank correlation coefficient, ρ. Statistical analyses were performed by BC on R (R Foundation for Statistical Computing, Vienna, Austria) Version 4.1.1 [22].

## 3 Results

Out of 58 students registered, 56 students participated to the formative 18-min OSCE with a SP (26 fourth-year student – 1st year of clinical clerkships) – females = 19 (73 %) and males = 7 (27 %); 30 sixth-year medical students (3rd year of clinical clerkships) – females = 25 (83 %) and males = 6 (17 %)). The case distribution was the following: 16 abdominal pain, 10 thoracic pain cases, 15 headache, and 15 dizziness cases (Appendix A). Sixteen interactions required mask wear because of the institutional requirements prevailing at the beginning of 2022.

Table 1 shows the perceived global empathy scores from students, SPs and the observer's perspectives, as well as measures of verbal empathy and non-verbal behavior using a dyadic coding system.

**Table 1:** Empathy scores.

Perspectives	Types of measurement	Score *N* = 56 Mean (SD)	Median (min-max)
Students' self-assessment	Global empathy score: ordinal scale (1−10)	7.30 (0.95)	7.00 (5.00–9.00)
SPs' assessment	Global empathy score: ordinal scale (1–10)	7.41 (2.13)	8.00 (2.00–10.00)
	Questionnaire: JSPPPE – P (5–35)	26.14 (6.90)	27.00 (9.00–35.00)
External observer’ s assessment	Global empathy score: ordinal scale (1–10)	5.89 (1.85)	6.00 (2.00–9.00)
	Questionnaire: JSPPPE – O (5–35)	23.05 (5.65)	25.00 (6.00–30.00)
Behavioral empathy	Verbal empathy (Verona Coding)	1.71 (1.32)	2.00 (0.00–6.00)
	Non-verbal behavior score (5–25) ⁎	18.83 (2.61)	19.00 (13.00–25.00)
	Non-verbal behavior score without facial expression (4–20)⁎⁎	15.20 (2.02)	15.00 (10.00–20.00)

⁎ The student's total score of non-verbal behavior is measured with the Verona Coding notation: eye contact, head movement, bodily posture and tone of voice which have all been evaluated on a Likert scale 1–5.

⁎⁎ The total score of non-verbal behavior without the facial expression item was included in the analyses because 16 of the students were wearing a protection mask because of Covid.

There was no or very weak evidence of a correlation between students' self-perceived global empathy score and either SPs' or the external observer's ratings (Table 2). Scores of global perceived empathy and Jefferson Scales scores (JSPPPE) showed high level of correlation, for both SPs and the observer respectively. There was also a moderate association between SP's and the observer's perceptions of empathy, independently from the measure used (global or JSPPE). Finally, both the external observer's perceptions of empathy and the SPs' perceptions of empathy were associated with verbal empathy (using the Verona dyadic coding).

**Table 2:** Correlation between self-assessment, standardized patients (SP)s' and observers (Obs)’ perceptions of empathy and verbal empathy.

	Student's global empathy score (1–10)	SP global empathy score (1–10)	Obs global empathy score (1–10)	JSPPPE – P (SP)	JSPPPE – O (Obs)	Verbal empathy (Verona)
Student's global empathy score (1–10)	1	0.16	0.18	0.15	0.24	0.22
SP global empathy score (1–10)		1	0.44**	0.88**	0.48**	0.27*
Obs global empathy score (1–10)			1	0.57**	0.89**	0.56**
JSPPPE – P (SP)				1	0.63**	0.34*
JSPPPE – O (Obs)					1	0.41**
Verbal empathy (Verona Coding)						1

**p value < .01, *p value < .05.

Non-verbal behavior showed the highest level of correlation with the external observer's perceptions of global empathy and weaker evidence with SPs' perceptions of global empathy and verbal empathy coded with the Verona dyadic coding system (Table 3). Head movement, bodily posture, and tone of voice were the non-verbal behaviors that correlated the most with verbal empathy (Verona coding) or perceived global empathy. Facial expression was also a non-verbal behavior associated with the observer's perceived global empathy.

**Table 3:** Correlation between different measures of empathy and non-verbal behavior.

	Non-verbal behavior (with facial expression) (5–25)	Non-verbal behavior (without facial expression) (5–25)	Non-verbal behavior eye contact (1–5)	Non-verbal behavior facial expression (1–5)	Non-verbal behavior head movement (1–5)	Non-verbal behavior bodily posture (1–5)	Non-verbal behavior tone of voice (1–5)
Self-perceived global empathy score (1–10)	0.03	0.03	−0.05	0.05	−0.10	0.17	0.14
SP global empathy score (1–10)	0.40*	0.39**	−0.05	0.28	0.35**	0.30*	0.35**
External observer empathy score (1–10)	0.68**	0.66**	0.27*	0.56**	0.52**	0.36**	0.51**
Verbal empathy (Verona Coding)	0.35*	0.38**	0.14	0.20	0.28*	0.27*	0.34**

**p value < .01, *p value < .05.

## 4 Discussion and conclusion

### 4.1 Discussion

The aim of this study was to explore the associations between perceived global empathy (students' self-perception, perceptions by SPs, and an external observer) and behavioral empathy including both verbal empathy and non-verbal behavior using a dyadic standardized coding system.

There was a lack of correlation between the self-perceived global empathy and the empathy assessed by SPs or an external observer. Studies investigating such associations in OSCE often relied on the Jefferson-student scale (JSPE-S), which does not measure self-perceived global or behavioral empathy during clinical encounters but self-perceived cognitive/affective empathy. Their findings varied. Conor et al. showed a weak but however significant correlation between SPs' assessment of empathy during OSCE and the JSPE-S score (ρ =0.23, *p* < .005), and no or much weaker evidence of a correlation between clinical examiners' scores of empathy during OSCE and JSPE-S scores (ρ =0.14, *p* < .08) [23]. Berg et al. evidenced a correlation between scores for students' self-report JSPE of empathy and scores of the JSPPPE SP (SP's perceptions of students' empathy) during a 10 station-OSCE taking place at the end of the third year (ρ = 0.19 *p* < .05) [9]. A randomized study examining the change of students' empathy after training showed that students' self-assessment did not change between both groups, while SPs and experts rated student's empathy as higher in the intervention group than in the control group [24].

We found significant associations between SPs' and external observer’ ratings, whatever the scale used (global or JSPPPE). These findings are in line with some studies on empathy [23] or communication skills [25,26] but differ from others showing higher SPs evaluations [27,28]. However, the timing of assessment of empathy may have mattered in one study [28]: SPs assessed students' performance immediately while external observers assessed videotaped sessions, and differences in rating were attributed to the difference of contexts of evaluation. The fact that SPs and external observers' scores were associated with verbal empathy in the present study suggests that they are valid and reliable estimators of empathy during OSCEs, even if SPs' assessment was immediate while external observer’ measures were done on videotaped sessions. Moreover, SPs and external observers' perspectives may be seen as more complementary than superposable, especially when the scoring process is accompanied by qualitative feedback. SPs may be the best placed to be able to assess students' interest and empathy in a specific context because they can feel the unseen, and directly experience the uniqueness of the student-patient relationship [29]. Complementarily, external observers may be more specific in giving feedback because they are able to relate their comments to the framework of reference used for teaching communication skills.

Finally, we could bring evidence that students' non-verbal behavior correlated not only with verbal empathy but also with both the external observer's and SPs' assessment of students' empathy. Head movement, bodily posture, and tone of voice were the non-verbal behaviors which showed the highest correlation rate with empathy. Studies have shown that non-verbal behavior correlates with both empathy and the patients' satisfaction [30,31]. Several studies have shown that the physician's gaze and his/her body orientation toward his/her patient are associated with a strong perception of clinical empathy, and a better understanding of the information given by the physician [[31], [32], [33]]. Vogel et al. suggested that non-verbal behavior is more important than verbal empathy, and is the primary channel for conveying emotions [14]. These findings indicate that non-verbal behavior is a valid indicator to assess the students' degree of empathy. In addition, the task of evaluating medical students' empathy could be fulfilled by SPs.

### 4.2 Innovation

A dyadic standardized coding system that codes both patients' expressions of emotions and students' responses to them using clear definitions offers a better and more reliable view of how empathy unfolds in real time than global scores or checklists. Little is known about the association between students', SPs', and observers' perceptions of behavioral empathy and measures of verbal empathy using a dyadic coding system. Even less is known about the link between these measures and non-verbal-behavior during OSCEs. The strength of our study relied on the inclusion of several perspectives regarding the assessment of empathy as well as the use of a dyadic approach of emotional communication. Our results indicate that the use of SPs and external observers are valid to measure behavioral empathy of medical students. Non-verbal behavior is highly correlated with verbal empathy. These finding suggest that behavioral empathy can be measured using SPs perceptions and that non-verbal behavior should be taken into consideration when assessing behavioral empathy.

There are however several limitations. First, the number of students involved in the study was small, with a higher percentage of female students than the gender distribution within our institution, and all students participated on a voluntary basis. In addition, all SPS were females. This situation could have introduced biases in our results since gender concordance is perceived as important for relationship issues. Satisfaction [34,35]. The external observer was an experienced teacher in communication who also assessed both non-verbal behavior and global empathy. We thus could not exclude that the coding processes may have interacted with each other, although they did not take place at the same time. However, the high inter-rater reliability of the non-verbal behavior coding supports the assumption of a low probability of such bias. Involving several external observers may be of interest, given the high levels of subjectivity inherent to communication skills assessment. Finally, the study took place in a formative OSCE setting and was based on encounters between students and SPs. This may have created an artificial environment which can alter the authenticity of the reactions of both parties in contrast when compared to a real workplace assessment. Finally, as the first formative OSCEs took place at the beginning of 2022, there were still institutional requirements regarding mask wear. Analysis of the students' facial expression was not possible for 16 interactions.

### 4.3 Conclusion

This study confirms the use of SPs and external observers are valid to measure behavioral empathy of medical students. Given the link between non-verbal behavior, behavioral empathy and patients' satisfaction, more attention should be paid to non-verbal communication in both teaching and assessment.

## CRediT authorship contribution statement

**A. Hepner:** Methodology, Investigation, Formal analysis, Conceptualization, Writing – original draft. **B. Cerutti:** Methodology, Formal analysis, Conceptualization, Writing – review & editing. **R. Lüchinger:** Methodology, Data curation, Conceptualization, Writing – review & editing. **N. Junod Perron:** Project administration, Methodology, Investigation, Funding acquisition, Formal analysis, Conceptualization, Writing – review & editing.

## Funding

The work was funded by the Geneva Faculty of Medicine (Mimosa Fund).

## Declaration of competing interest

The authors declare that they have no known competing financial interests or personal relationships that could have appeared to influence the work reported in this paper.

## Figures and Tables

**Table:** 

Case	Worries	SP 1	SP 2	SP 3	SP 4	SP 5
30–35-year-old patient complaining on intermittent **abdominal pain** for the last 2 months	Fear of endometriosis Professional stress in a new job	16	0	0	0	0
30–35-year-old patient complaining on intermittent **thoracic pain** for the last 3 weeks	Fear of a heart problem Professional stress in a new job	0	5	0	5	0
30–35-year-old patient complaining on intermittent **headache** for the last 2 months	Fear of aneurysm rupture Professional stress in a new job	0	0	8	0	7
30–35-year-old patient complaining on intermittent **diziness** for the last 2 months	Fear of multiple sclerosis Professional stress in a new job	0	0	0	15	0

**Figure:**
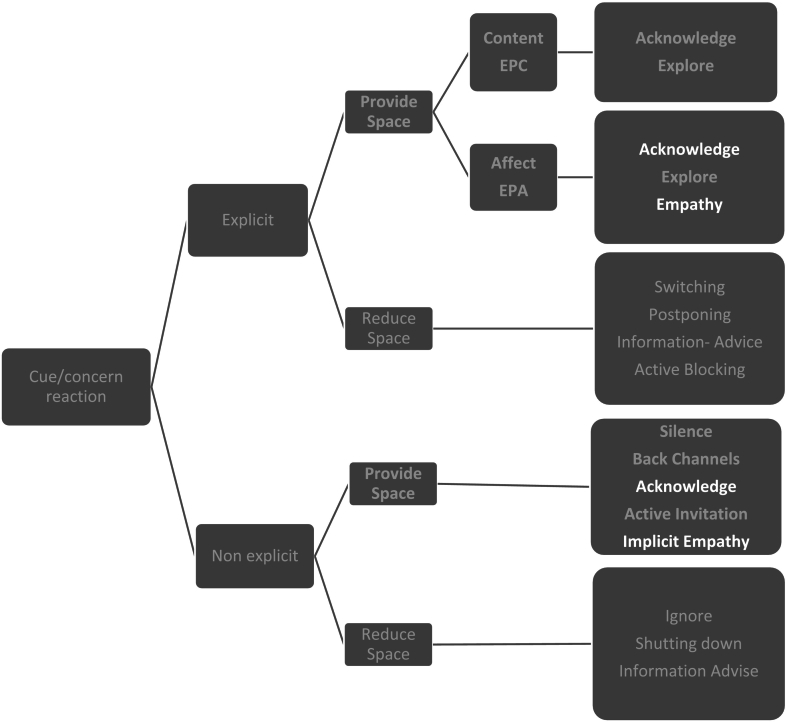


## References

[1] van Dulmen S., van den Brink-Muinen A. (2004). Patients’ preferences and experiences in handling emotions: a study on communication sequences in primary care medical visits. Patient Educ Couns.

[2] Derksen F., Bensing J., Lagro-Janssen A. (2013). Effectiveness of empathy in general practice: a systematic review. Br J Gen Pract.

[3] Yang N., Xiao H., Wang W., Li S., Yan H., Wang Y. (2018). Effects of doctors’ empathy abilities on the cellular immunity of patients with advanced prostate cancer treated by orchiectomy: the mediating role of patients’ stigma, self-efficacy, and anxiety. Patient Prefer Adherence.

[4] Sulzer S.H., Feinstein N.W., Wendland C.L. (2016). Assessing empathy development in medical education: a systematic review. Med Educ.

[5] Kane G.C., Gotto J.L., Mangione S., West S., Hojat M. (2007). Jefferson scale of patient’s perceptions of physician empathy: preliminary psychometric data. Croat Med J.

[6] Davis M.H. (1983). Measuring individual differences in empathy: evidence for a multidimensional approach. J Pers Soc Psychol.

[7] Stepien K.A., Baernstein A. (2006). Educating for empathy. A review. J Gen Intern Med.

[8] Hojat M., Mangione S., Nasca T.J., Cohen M.J.M., Gonnella J.S., Erdmann J.B. (2001). The Jefferson scale of physician empathy: development and preliminary psychometric data. Educ Psychol Meas.

[9] Berg K., Majdan J.F., Berg D., Veloski J., Hojat M. (2011). Medical students’ self-reported empathy and simulated patients’ assessments of student empathy: an analysis by gender and ethnicity. Acad Med.

[10] Bussenius L., Kadmon M., Berberat P.O., Harendza S. (2022). Evaluating the global rating scale’s psychometric properties to assess communication skills of undergraduate medical students in video-recorded simulated patient encounters. Patient Educ Couns.

[11] Hodges B., McIlroy J.H. (2003). Analytic global OSCE ratings are sensitive to level of training. Med Educ.

[12] Irving P., Dickson D. (2004). Empathy: towards a conceptual framework for health professionals. Int J Health Care Qual Assur Inc Leadersh Health Serv.

[13] LaNoue M.D., Roter D.L. (2018). Exploring patient-centeredness: the relationship between self-reported empathy and patient-centered communication in medical trainees. Patient Educ Couns.

[14] Vogel D., Meyer M., Harendza S. (2018). Verbal and non-verbal communication skills including empathy during history taking of undergraduate medical students. BMC Med Educ.

[15] Brown L.E., Chng E., Kortlever J.T.P., Ring D., Crijns T.J. (2023). There is little or no association between independently assessed communication strategies and patient ratings of clinician empathy. Clin Orthop Relat Res.

[16] Qualtrics XM: The leading Experience Manamgenet Softwre (2005).

[17] Del Piccolo L., de Haes H., Heaven C., Jansen J., Verheul W., Bensing J. (2011). Development of the Verona coding definitions of emotional sequences to code health providers’ responses (VR-CoDES-P) to patient cues and concerns. Patient Educ Couns.

[18] Zimmermann C., Del Piccolo L., Bensing J., Bergvik S., De Haes H., Eide H. (2011). Coding patient emotional cues and concerns in medical consultations: the Verona coding definitions of emotional sequences (VR-CoDES). Patient Educ Couns.

[19] Silverman J., Kurzt S., Draper J. (2013).

[20] Blanch-Hartigan D., Ruben M.A., Hall J.A., Schmid Mast M. (2018). Measuring nonverbal behavior in clinical interactions: a pragmatic guide. Patient Educ Couns.

[21] Behavioral coding- Event logging software (2025). The Observer XT. https://www.noldus.com/observer-xt.

[22] (2025). R: The R Project for Statistical Computing. https://www.r-project.org/.

[23] O’Connor K., King R., Malone K.M., Guerandel A. (2014). Clinical examiners, simulated patients, and student self-assessed empathy in medical students during a psychiatry objective structured clinical examination. Acad Psychiat: J Am Assoc Direct Psychiat Res Train Assoc Acad Psychiat.

[24] Wundrich M., Schwartz C., Feige B., Lemper D., Nissen C., Voderholzer U. (2017). Empathy training in medical students - a randomized controlled trial. Med Teach.

[25] Humphrey-Murto S., Smee S., Touchie C., Wood T.J., Blackmore D.E. (2005). A comparison of physician examiners and trained assessors in a high-stakes OSCE setting. Acad Med.

[26] Shirazi M., Labaf A., Monjazebi F., Jalili M., Mirzazadeh M., Ponzer S. (2014). Assessing medical students’ communication skills by the use of standardized patients: emphasizing standardized patients’ quality assurance. Acad Psychiat: J Am Assoc Direct Psychiat Res Train Assoc Acad Psychiat.

[27] Turner T.R., Scerbo M.W., Gliva-McConvey G.A., Wallace A.M. (2016). Standardized patient encounters: periodic versus postencounter evaluation of nontechnical clinical performance. Simul Healthc.

[28] Wollney E.N., Vasquez T.S., Stalvey C., Close J., Markham M.J., Meyer L.E. (2023). Are evaluations in simulated medical encounters reliable among rater types? A comparison between standardized patient and outside observer ratings of OSCEs. PEC Innov.

[29] Zoppi K., Epstein R.M. (2002). Is communication a skill? Communication behaviors and being in relation. Fam Med.

[30] Klockner C.C., Gerbase M.W., Nendaz M., Baroffio A., Junod N.P. (2022). Relationship between self-reported cognitive and behavioural empathy among medical students. Patient Educ Couns.

[31] Larson E.B., Yao X. (2005). Clinical empathy as emotional labor in the patient-physician relationship. JAMA.

[32] Brugel S., Postma-Nilsenova M., Tates K. (2015). The link between perception of clinical empathy and nonverbal behavior: the effect of a doctor’s gaze and body orientation. Patient Educ Couns.

[33] Mehrabian A., Ferris S.R. (1967). Inference of attitudes from nonverbal communication in two channels. J Consult Psychol.

[34] Schmid Mast M., Hall J.A., Roter D.L. (2007). Disentangling physician sex and physician communication style: their effects on patient satisfaction in a virtual medical visit. Patient Educ Couns.

[35] Garcia J.A., Paterniti D.A., Romano P.S., Kravitz R.L. (2003). Patient preferences for physician characteristics in university-based primary care clinics. Ethn Dis.

